# Insights Into the Helical Shape Complex of *Helicobacter pylori*

**DOI:** 10.3389/fmicb.2022.929194

**Published:** 2022-08-24

**Authors:** Sven Holtrup, Maximilian Greger, Benjamin Mayer, Mara Specht, Barbara Waidner

**Affiliations:** ^1^LOEWE Center for Synthetic Microbiology, Marburg, Germany; ^2^Department of Biochemistry and Chemistry, Philipps University of Marburg, Marburg, Germany

**Keywords:** bactofilin, single-molecule tracking, *Helicobacter pylori*, cell shape, structured illumination microscopy

## Abstract

One important factor that promotes the colonization of the upper digestive system of the human pathogen *Helicobacter pylori* is its helical cell shape. The bacteria cell shape is predominantly defined by its peptidoglycan cell wall. In rod-shaped species, PG synthesis is mediated by two dynamic molecular machines that facilitate growth along the perpendicular axis and the septum, called the elongasome and the divisome, respectively. Furthermore, many bacteria evolved additional mechanisms to locally change PG synthesis patterns to generate diverse cell shapes. Recent work characterizing cell shape mutants of *Helicobacter pylori* revealed a novel mechanism for the generation of a twisted helix from a rod, including PG-modifying enzymes as well as additional proteins such as the bactofilin homolog CcmA or the membrane proteins Csd5 and Csd7. In this study, we investigate the localization and dynamics of CcmA and Csd7 using live-cell imaging. We also address the question of how these change in the presence or absence of the putative interaction partners.

## Introduction

Bacterial cells are usually associated with characteristic morphology and size, and many plausible models regarding the functional significance of cell morphology have emerged (Young, 2006; Yang et al., 2016). The ε-proteobacterium *Helicobacter pylori* is known for its colonization of the human stomach where it potentially causes gastric inflammations leading to ulcers and gastric cancer (Marshall and Warren, 1984). Among other pathogenicity factors, its typical cell shape has been described to enhance efficient stomach colonization (Bonis et al., 2010; Sycuro et al., 2010). Studies on the molecular basis of *H. pylori* have shed light on a novel mechanism of cell shape control as reviewed in Salama (2020). As such, a set of peptidoglycan endopeptidases, as well as cytoskeletal proteins, has been identified to act in concert and promote helical cell shape by locally relaxing and cross-linking the PG sacculus (Bonis et al., 2010; Sycuro et al., 2010, 2012). Factors predicted to work within this complex are the cell shape determining proteins Csd1/2, Csd5, and Csd7 as well as the bactofilin homolog CcmA and the PG precursor synthase MurF (Blair et al., 2018; Yang et al., 2019). The M23-type metallopeptidase Csd1 forms a heterodimer with its enzymatically inactive counterpart Csd2 which cleaves PG tetra-penta cross-links (An et al., 2016). Further factors found to be involved in helical shape generation are Csd3/HpdA, Csd4, and Csd6 (Sycuro et al., 2013). Csd5 is a single-pass inner membrane protein that has a short cytoplasmatic N-terminal domain which was found to interact with CcmA and MurF and a C-terminal SH3 domain which interacts with the PG (Blair et al., 2018). Another direct connection between cytoplasmatic factors and the periplasm is the multi-transmembrane domain protein Csd7, which was found to interact with Csd2 in the periplasm and was found to co-purify with CcmA and MurF in co-immunoprecipitation, respectively (Yang et al., 2019). The combination of these results in *H. pylori* cells suggests the existence of one or more the so-called Shapeosome complexes, for which a schematic model was postulated and presented in Salama (2020).

Bactofilins resemble a widespread family of bacterial polymer-forming proteins. Interestingly, some examples have recently been described in the eukaryotic stramenopiles clade (Deng et al., 2019). Bactofilins are defined by a central beta-helical domain (DUF583), which enables polymerization into filaments or 2D crystalline sheets based on hydrophobic interactions. This central domain is usually flanked by mainly unstructured N- and C-terminal regions that are thought to mediate protein–protein interactions. Filaments are largely insensitive to pH, salt, or chelating agents (Koch et al., 2011; Deng et al., 2019; Holtrup et al., 2019). Diverse functions have been described for bactofilins, ranging from directing PG modifications (Kühn et al., 2010; Sycuro et al., 2010) to chromosome segregation (Lin et al., 2017) to flagella synthesis and motility (El Andari et al., 2015). In the stalked budding bacterium *C. crescentus*, the two homologs BacA and BacB cooperate to form a sheet-like structure that mediates the localization of a PG synthesis to the stalk (Kühn et al., 2010). Analogously, in its close relative *Asticaccaulis biprosthecum*, the bactofilin homolog BacA is recruited by the morphogen SpmX to organize zonal PG synthesis at the stalks (Caccamo et al., 2020). Interestingly, bactofilin genes in both organisms are within an operon with M23-type endopeptidases, as *csd1/2* and *ccmA*. This is also true for the *Thermus thermophilus* homolog TtBacA, of which cryo-EM data gave insights into the structure of bactofilin filaments, demonstrating that monomers polymerize in a head–head-like manner (Deng et al., 2019).

Despite an increasing knowledge on bactofilins, the role of CcmA in generating *H. pylori* helical shape is still under the investigation. According to the latest model, a core of the putative shape complex is likely to be formed by the membrane-spanning non-enzymatic protein Csd7, which interacts with Csd2 in the periplasm, and may be bound to the membrane-associated CcmA, which in turn is thought to interact directly with the inner membrane proteins MurF and Csd5. However, it is still unclear how these proteins work together to promote cell shape. Subcellular localization is one of the most important aspects in determining protein function. For this reason, we have previously shown by immunofluorescence that the *H. pylori* bactofilin CcmA localizes as subcellular accumulations in varying numbers adjacent to the cell membrane (Holtrup et al., 2019). A recent study, also using immunofluorescence, confirmed this finding and showed that the *H. pylori* bactofilin of strain LSH100 is present in cells as numerous spots with the preference of the major axis. Here, the authors propose a model where CcmA and MreB promote PG synthesis at areas of increased positive and negative Gaussian curvatures, respectively (Taylor et al., 2020).

In this study, we focus on the subcellular *in vivo* localization and dynamics of the bactofilin CcmA in two different *H. pylori* strains using fluorescent fusions of CcmA at the native gene locus. This allowed us to perform epifluorescence, super-resolution microscopy, and single-particle (molecule) tracking (SPT/SMT) (Rösch et al., 2018). We also addressed the question of whether the absence of the putative interaction partners Csd5 and Csd7 changes the dynamics of CcmA and shows Csd7 *in vivo* localization and dynamics both in the presence and in the absence of CcmA.

## Results

### The *H. pylori* Bactofilin Gfp Fusion Can Functionally Replace the Native Bactofilin in Strains 26695 and G27

**FIGURE 1:**
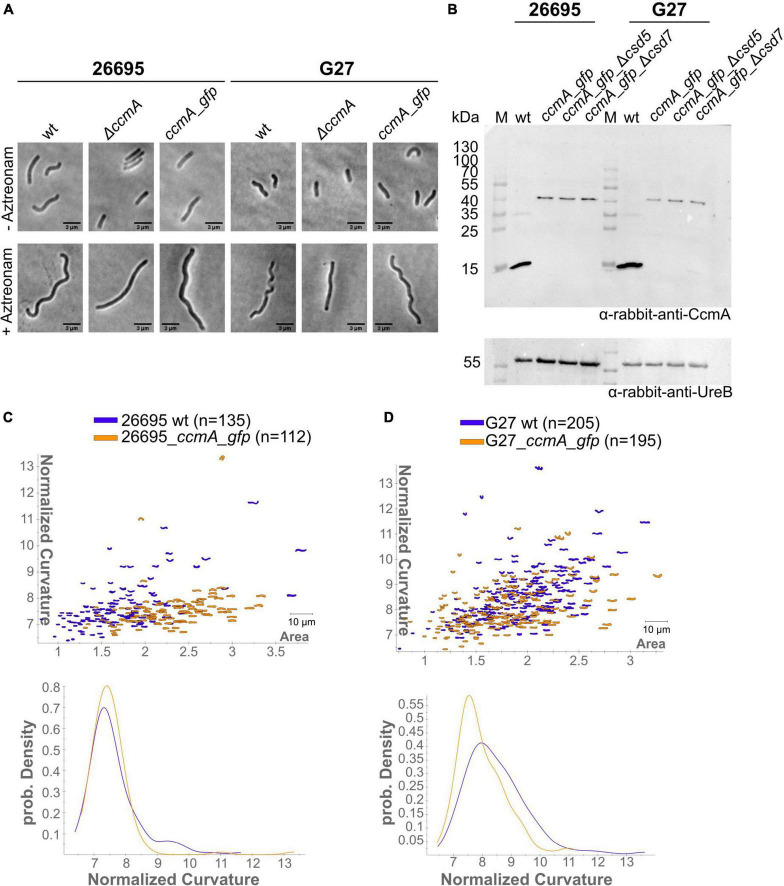
**(A)** Exemplary phase-contrast images illustrating the effect of the insertion of *ccmA_gfp* at the native locus on the cell shape of *H. pylori* 26695 and G27. For a better comparison, filamentous growth was induced by treating cells with Aztreonam. CcmA_Gfp cells are slightly less curved as compared to the wt but clearly not as straight as the *ccmA* knockout. **(B)** Western blot of *H. pylori* strains was used in this study. The membrane was first probed with CcmA-specific antibodies. Afterward, the membrane was stripped and again probed with UreB-specific antibodies, serving as a loading control. **(C)** Comparison of 26695 wt and 26695_*ccmA_gfp* by principal component analysis and probability density plot of normalized curvature. **(D)** Comparison of G27 wt and G27_*ccmA_gfp* by principal component analysis and probability density plot of normalized curvature.

Subcellular localization of the *H. pylori* bactofilin has previously been described by us and others using immunofluorescence. By that, it was shown that CcmA preferentially localizes to the areas of increased positive Gaussian curvature and that altered polymerization properties lead to the loss of helical shape (Taylor et al., 2020). To analyze *in vivo* dynamics of *CcmA*, we generated strains in which a *C-terminal* Gfp-tagged version of *ccmA* was located under the native promotor in the chromosome. *H. pylori* isolates display diverse morphologies (Martinez et al., 2016). Since a major question is how CcmA affects cell shape, we used the laboratory strains G27 and 26695, as both strains are well characterized and exhibit a different morphological plasticity, with G27 cells being a twisted helix, whereas 26695 cells have predominantly curved rod morphology. Since the loss of CcmA leads to a drastic defect in cell shape, we were able to verify the influence of the Gfp-tag on our *ccmA*_*gfp* strain by qualitative and quantitative shape analyses. For qualitative analysis, we acquired phase-contrast images of both wt strains and compared them with their respective *ccmA_gfp* derivatives (Figure 1A). Qualitative comparisons of these images gave the impression that both *ccmA_gfp* strains showed similar morphology to the wild type in both strains, with tendency toward a slightly less curved shape (Figure 1A, upper panels). To more accurately assess cell shape and highlight potential shape defects, we induced filamentous growth by treatment with the drug aztreonam (Figure 1A, lower panels). Thereby, the effects on the spiral cell shape were highlighted, thus facilitating the interpretation of the images. We confirmed the correct size of our fusion product, as well as semiquantitatively monitored the protein amount by western blot analysis over the growth phases (Figure 1B), thus demonstrating that our Gfp fusion did not show degradation of CcmA and Gfp in either strain (Figure 1B and Supplementary Figure 1). However, the signal appears to be somewhat weaker in the Gfp fusion cells. This could partially result from the increase in molecular weight and associated transfer effects during blotting; however, we cannot rule out that protein levels are affected. Further, no change in signal intensity during the cell cycle could be detected in wt and *ccmA_gfp* fusion strains, as normalization to the loading control (UreB) demonstrated (Supplementary Figure 1).

For quantitative analysis of the phase-contrast images, the CellTool software described in the study of Pincus and Theriot (2007) was used. In this procedure, the contours of the cells were extracted from the phase-contrast images and plotted by area against curvature normalized by cell length. By comparing 26695 wt cells to 26695 *ccmA_gfp* cells, both cell populations share a large portion of similarly shaped cells. However, 26695 wt cells additionally exhibit a small population of cells with higher curvature which is not present in *ccmA_gfp* cells (Figure 1C). This observation is more pronounced in strain G27 (Figure 1D). In summary, our results show that the distribution of CcmA_Gfp cells in both strains strongly overlaps with that of the corresponding wt cells. In both cases, however, the wt cells show a slightly broader distribution toward higher curvature, reflecting the rather small influence of the protein fusion. Taken together, we concluded that the *ccmA_gfp* fusions in both strains are capable of replacing most of the wt bactofilin function. However, some limitations due to spherical effects could not be excluded.

### CcmA Is Localized *in vivo* to Defined Foci at the Cell Membrane

**FIGURE 2:**
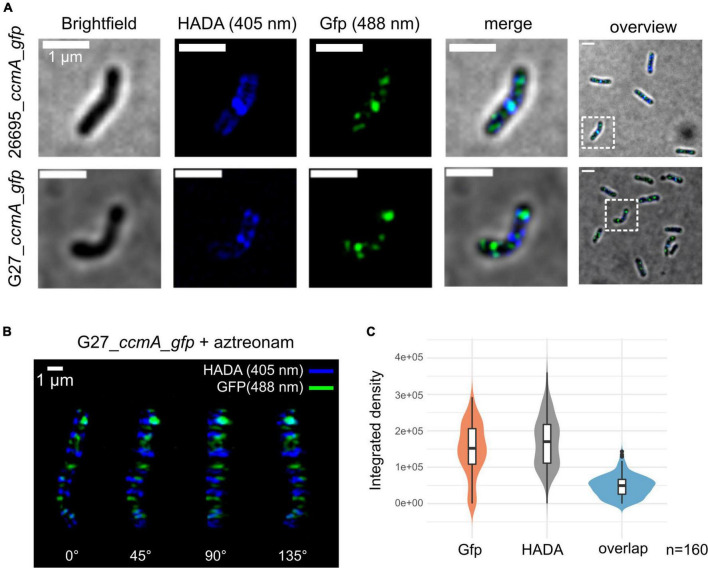
Structured illumination microscopy of 26695_ccmA_gfp and G27_ccmA_gfp. Exponentially growing cells were pulse-labeled with HADA and images were reconstructed from three rotation angles **(A)** Brightfield, HADA (blue) and GFP (green) channels of exemplary 26695_ccmA_gfp (upper lane) and G27_ccmA_gfp (lower lane) cells. **(B)** 3D-view of aztreonam-treated G27_ccmA_gfp cell containing multiple growth rings. **(C)** Violin plot of integrated density distributions of the HADA- and Gfp signals compared to overlapping regions of 26695_ccmA_gfp cells.

To get detailed insights into the subcellular localization of CcmA, we performed structured illumination microscopy (SIM) with exponentially growing cells of our generated *ccmA_gfp* fusion strains using multi-color SIM mode with three different excitation wavelengths and three rotations (Figure 2A). CcmA_Gfp was found to localize to the inner membrane in both strains as small foci as shown by projections of reconstructed Z-stacks (Figure 2A).

Because the current model describes CcmA as the part of a dynamic PG-modifying complex preferentially localized at positive super curvature (Taylor et al., 2020), we were interested in whether these patches would localize to the areas of increased peptidoglycan insertion. Therefore, we visualized the zones of increased PG synthesis by pulsed incubation with the fluorescently labeled D-alanine derivative HADA (3-[[(7-hydroxy-2-oxo-2H-1-benzopyran-3-yl)carbonyl]amino]-D-alanine). Initial inspection indicated that the CcmA_Gfp signal, although broadly distributed across the cell, was often present close to the HADA signal but did not appear to overlap with it (Figure 2A). To facilitate visualization and analysis of this pattern, we induced filamentous growth by treatment with aztreonam (Figure 2B). Because aztreonam inhibits Pbp2A at the septum and thus blocks septation, treatment results in multiple growth rings in elongated cells. This points out the observed pattern illustrated by the projection of the 3D reconstructed filamentous cells G27_ccmA_gfp (Figure 2B). Also, the signals from CcmA_Gfp and HADA appear to be only close to each other, without being colocalized (Figure 2B). We complemented this observation with a quantification of the signal overlap by subtracting Z-projections of individual cells (*n* = 160) from three independent replicates and measuring the respective integrated densities. As visualized by violin plots, we found only very few signals to overlap (Figure 2C). Furthermore, to examine the correlation of the two signals, we used the Pearson correlation coefficient (PCC), which is a measure of the linear relationship between two sets of data. The signal correlation was performed using the Fiji (Schindelin et al., 2012) plugin JACoP (Bolte and Cordelières, 2006). This yielded a Pearson correlation coefficient of *r* = 0.103 and Manders coefficients of M1 = 0.007 (proportion of Gfp signal overlapping with HADA proportion) and M2 = 0.004 (HADA proportion overlapping with Gfp proportion), indicating that the two signals are neither correlated nor overlapping.

### CcmA_Gfp Molecules Form Clusters Near the Cell Membrane Distributed Along the Cell, Occasionally Jumping From One Cluster to Another

**FIGURE 3:**
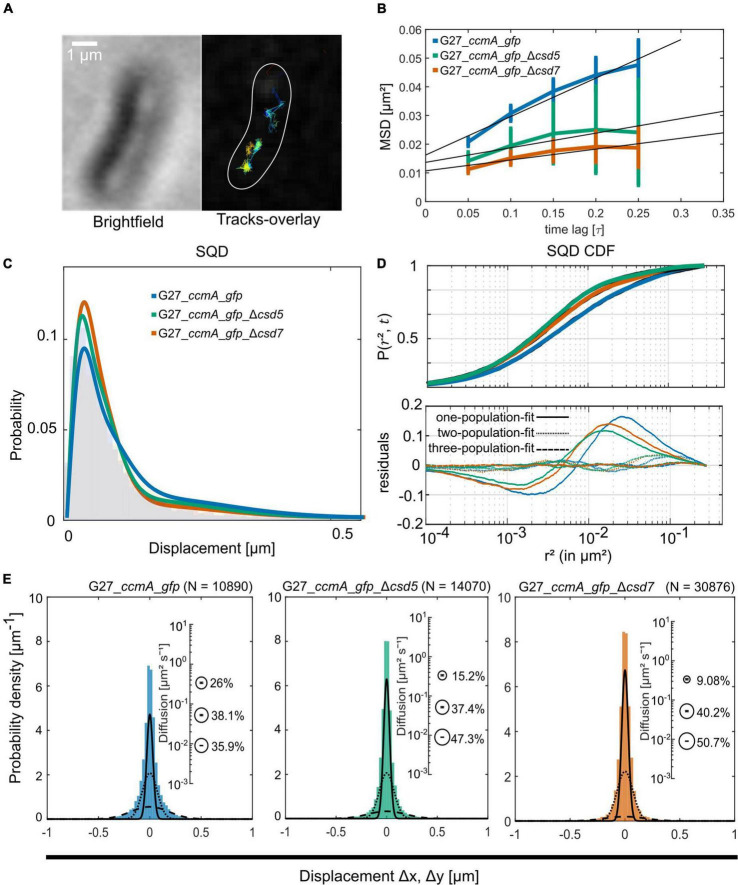
Single-molecule dynamics of CcmA_Gfp in wt, Δcsd5, and Δcsd7 background. **(A)** Brightfield image of an example cell and respective CcmA_Gfp tracks. Scale bar = 1 μm. **(B)** Mean-squared displacement of single-molecule tracks of CcmA_Gfp in different strain backgrounds, plotted against the time lag. **(C)** Density plot illustrating the probability for different squared displacement lengths for CcmA_Gfp. **(D)** The cumulative distribution function of CcmA_Gfp squared displacements. The probability for a molecule to stay within a certain radius *r*^2^ at a certain time *t* is plotted against the radius. The residue plot in the lower panel illustrates the differences of the applied models (single, double, or triple fit) to the data. **(E)** Probability densities of single-molecule displacements fitted by Gaussian-mixture models. Population sizes estimated from a triple-fit model are illustrated as bubble plots. We observed a slight decrease of CcmA_Gfp single-step distances in the deletion strains as compared to wt background.

To study the localization of CcmA in terms of dynamics, we used the established single-molecule tracking approach (SMT) of exponentially growing cells. In this approach, cells are first bleached with a slim-field laser until individual fluorophore movements can be observed, which has been shown to be a powerful tool for analyzing dynamic processes in living cells [reviewed in Rösch et al. (2018)]. It was shown that freely diffusing Gfp molecules can only be tracked accurately with very fast acquisition rates (e.g., 5 ms; Stracy et al., 2014; Schibany et al., 2018). Since we wanted to focus on slower diffusing molecules and exclude these background activities from our analysis, we set our acquisition rate at 50 ms. Using this approach, we confirmed that the individual CcmA_Gfp molecules were localized in clusters near the membrane along the cell. Qualitative motion analysis using the Fiji (Schindelin et al., 2012) plugin TrackMate (Tinevez et al., 2017) revealed a picture of signal clusters (Figure 3A), in which individual molecules occasionally jumped from one cluster to another. To characterize these arbitrarily shaped clusters, we analyzed the data using the density-based spatial clustering of applications with noise (DBSCAN) algorithm, which is commonly used to quantitatively assess subcellular assemblies. Clusters are defined based on the search radius (R) and the minimum number of single-molecule localizations (SMLs) within that radius (N). As such, the DBSCAN algorithm was applied to the tracking data obtained from the same movies but with the Fiji package TrackMate (Tinevez et al., 2017), and we used the R packages DBSCAN (Hahsler et al., 2019) and fpc (Hennig, 2020) for the analysis. Using the identical parameters, an average value of 6.84 ± 2.65 and 6.91 ± 2.64 clusters per cell was obtained for strains 26695_*ccmA_gfp* and G27_*ccmA_gfp*, respectively (Supplementary Figure 3). In addition, we also wanted to get a picture of the dynamics within the cell-shaping complex. Therefore, we focused on the two proteins potentially interacting with CcmA, Csd5, and Csd7 (Blair et al., 2018). Csd7 is a multi-transmembrane domain protein that is thought to act as a link between cytoplasmic factors and the periplasmic LytM domain of the endopeptidase Csd1. Csd5 is a single membrane-bound protein with a periplasmic SH3b domain and a short cytoplasmic appendage that has been shown to interact directly with CcmA (Blair et al., 2018). Therefore, we generated deletion strains of *csd5* and *csd7* in the *ccmA_gfp* background of both wt strains. First, we also examined the CcmA clusters in these strains using the DBSCAN algorithm. The result showed that the average number of clusters was almost the same for all strains (Supplementary Figure 3).

For further SMT analysis, bleaching curves were generated in the G27 strains and the movies were cropped to single-molecule level. Detection of single-molecule tracks was performed with u-track (Jaqaman et al., 2008), and cell edges were labeled with Oufti (Paintdakhi et al., 2016). These data were combined in the SMTracker software as described in the study of Rösch et al. (2018). All single-molecule data analyses were done using SMTracker (Rösch et al., 2018), which uses *R*^2^ as a measure for goodness of fit and several statistical tests (Kolmogorov–Smirnov goodness-of-fit, null hypothesis significance) to determine whether acquired distributions of molecule movements can be explained by single or multiple fractions.

A common approach to describe the molecular motion of individual particles is the mean square displacement (MSD). Here, molecular 2D displacements are averaged over several steps (τ). For our analysis, only continuous tracks of at least five steps were considered. As a result, we obtained an overall diffusion constant of *D* = 0.044 μm^2^/s for CcmA_Gfp in the presence and 0.02 μm^2^/s and 0.019 μm^2^/s in the absence of Csd5 and Csd7, respectively (Figure 3B and Table 1). Thus, the results showed a decrease in the overall diffusion of CcmA_Gfp in the absence of the putative interaction partners Csd5 and Csd7.

**TABLE 1:** Summary of CcmA_Gfp single-molecule dynamics.

Strain	G27_*ccmA_gfp*	G27_*ccmA_gfp_*Δ *csd5*	G27_*ccmA_gfp_*Δ *csd7*
**MSD**			
#Tracks	806	835	1662
*D xy* (μm^2^ s^–1^)	0.0440	0.0200	0.0130
**SQD**			
Static *D* (μm^2^ s^–1^)	0.00715	0.0048	0.00595
Slow-mobile *D* (μm^2^ s^–1^)	0.0376	0.0251	0.0252
Mobile *D* (μm^2^ s^–1^)	0.303	0.243	0.255
Static fraction (%)	26.5	21.8	26.4
Slow-mobile fraction (%)	43.9	55.2	57
Mobile-fraction (%)	29.6	23.1	16.6
**GMM**			
Static *D* (μm^2^ s^–1^)	0.009	0.009	0.009
Slow-mobile *D* (μm^2^ s^–1^)	0.052	0.052	0.052
Mobile *D* (μm^2^ s^–1^)	0.34	0.34	0.34
Static Fraction (%)	36 ± 0.58	47 ± 0.7	51 ± 0.77
Slow-mobile fraction (%)	38 ± 0.74	37 ± 0.55	40 ± 0.52
Mobile-fraction (%)	26 ± 0.66	15 ± 0.62	9.1 ± 0.65

Next, we analyzed the diffuse behavior at subpopulation levels. Therefore, we applied a squared displacement (SQD) analysis that scores the probability density of the squared *x*/*y*-displacements of certain molecules (Figure 3C). The cumulative distribution function (CDF) of these squared displacements (*r*^2^) represents the probability P (r^2^, t) of a molecule remaining in a circle of radius r in time t. To determine whether the recorded distributions of molecular motions could be represented by single or multiple fractions, the SMTracker software used *R*^2^ as the goodness of several statistical tests (Kolmogorov–Smirnov goodness-of-fit, null hypothesis significance) (Figure 3D). To avoid overfitting, the approximate number of diffuse groups is determined using the Bayes information criterion. The bottom panel of Figure 3D shows the differences (“residuals”) between the modeled data and the measured values with respect to random diffusion, represented by the baseline (“0”). The smallest deviation from our model occurred when using a triple fit, indicating that this best describes the situation of our fusion protein. However, it should be noted that the SMTracker software does not consider higher-order adjustments. In brief, the static population of G27_*ccmA_gfp* represents 26.5% of all captured movements (*D* = 0.018 μm^2^/s), the slow-mobile 43.9% (*D* = 0.038 μm^2^/s), and the mobile fraction 29.6% (*D* = 0.303 μm^2^/s). In the absence of Csd5 or Csd7, we observe that the mobile population shifts to the smaller ones, which is consistent with the MSD analysis (Table 1). In addition, this analysis also suggests an increase in shorter tracks for CcmA_Gfp in *csd5*- and *csd7*-deficient cells. Individual analysis of three replicates showed only minor day-specific differences and further validated the analysis of the datasets (Supplementary Figure 3). Another commonly used strategy for subpopulation analysis is the Gaussian mixture model (GMM). Unlike the SQD method, the GMM is based on the probability that molecules take a number of steps. The Kolmogorov–Smirnov goodness-of-fit (KS GoF) implemented in SMTracker 2.0 rejected two and one population fits, whereas three populations were accepted for the three datasets (Figure 3E and Table 1). Both the fraction sizes and the corresponding diffusion coefficients derived from this GMM analysis show a decrease in the fast fraction whereas the static fraction size increases in *csd5*- and *csd7-*deficient cells, supporting the previous results of the SQD and MSD analysis (refer to bubble bots Figure 3D and Table 1). However, the observed effect on the CcmA diffusion of the two missing proteins is rather small.

### The Polymeric State of CcmA Is Represented by the Static Population

**FIGURE 4:**
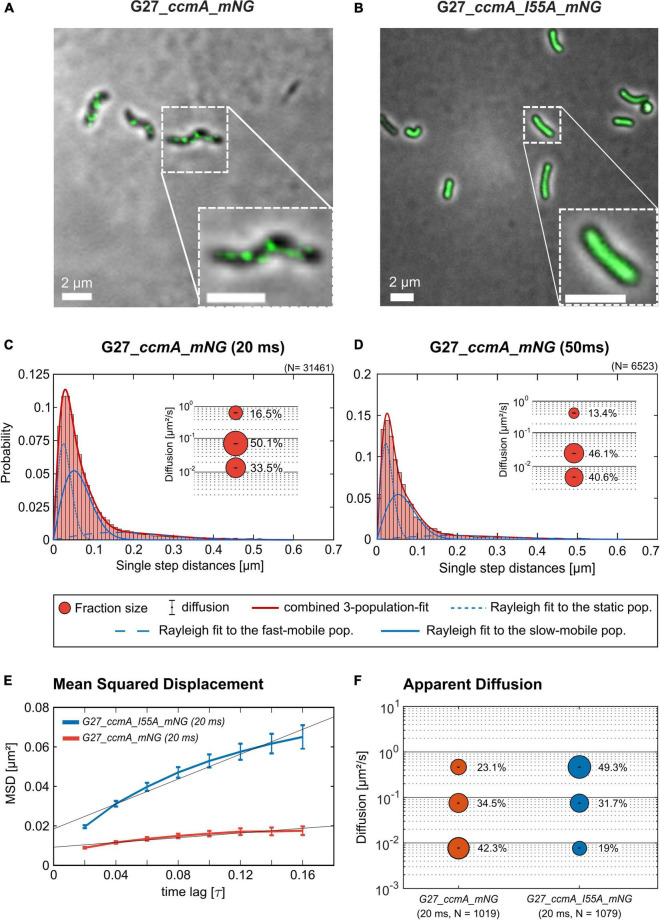
Structured illumination microscopy imaging of CcmA_mNG **(A)** and CcmA_I55A_mNG **(B)** in G27 at exponential phase, scale = 2 μm. Polymeric CcmA forms distinct foci at the membrane as shown by Z-projection while its mutated derivate CcmA_I55A is distributed equally over the cell (microscope settings mNG: 488 nm laser, 10.0%, exp. time: 20 ms, 3 rotations, EMCCD gain: 50.0). **(C,D)** Squared displacement analysis (SQD) of CcmA_mNG with an exposure time of 20 **(C)** and 50 ms **(D)** per frame of single-molecule tracks as obtained from slim-field bleaching microscopy. Diagrams display the probability for each detected jump distance with non-simultaneous SQD curve fitting. Data acquired with 50-ms exposure time represent one replicate. **(E)** Mean-squared displacement curves with an aligned linear fitting to the first 9 time lags showing a general diffusion regardless of subdiffusion. The MSD analysis demonstrates that the inhibited polymerization of CcmA_I55A results in an overall faster movement. **(F)** Apparent diffusion analysis of CcmA_mNG and CcmA_I55A_NG in G27 of single-molecule tracks obtained from slim-field bleaching microscopy. Bubble plots show the estimated population and diffusion coefficients as identified by simultaneous APPD curve fitting. Data derived from at least three biological triplicates with an exposure time of 20 ms per frame. CcmA_I55A_mNG (126 cells, *N* = 1,079 tracks) exhibited a strong shift to the mobile population as compared to CcmA_mNG (113 cells, *N* = 1,019 tracks) while the slow-mobile population remain unaffected, suggesting a functional state represented by the static population.

As another control for our CcmA fusion, we generated a CcmA_mNG fusion as mNeonGreen (mNG) was reported as the brightest monomeric green or yellow fluorescent protein (Shaner et al., 2013). Furthermore, mNG exhibits increased photostability and a shorter maturation time (Shaner et al., 2013). In addition, we placed a linker between the bactofilin and the fluorophore to address concerns about potentially impaired interaction of the individual CcmA monomers (please refer to Supplementary Figure 4 for a comparison of the two structures). Also, we created a mNG fusion of the point mutant CcmA_I55A, for which polymerization defects and a diffuse *in vivo* localization as foci were reported (Taylor et al., 2020). The correct size and stability of both fusion products were confirmed by western blot analysis (Supplementary Figure 5). Structural illumination microscopy (SIM) images of exponentially growing cells confirmed the helical shape of the CcmA_mNG (Figure 4A) fusion and exhibited a similar *in vivo* localization as compared to CcmA_Gfp (Figure 1A). No preference for areas of positive or negative curvature was observed (Figures 3D, 4A visualization in Supplementary Movie 1), which we also not observed for CcmA_Gfp. As expected, the point mutation showed a straight phenotype, and *in vivo* localization analysis revealed a signal distributed throughout the cell without any preferences (Figure 4B). Furthermore, the polymeric state of CcmA is thought to be the active functional form (Taylor et al., 2020). In addition, also, the observed dynamics of the CcmA_mNG fusion using the SQD analysis represented the results similar to the CcmA_Gfp fusion (Figure 4D). As SMT analysis of the non-polymerizing mutant allows us to distinguish which of the observed subpopulations represents the active functional fraction, we subsequently performed SMT analyses with both variants. Based on the observed signal distributed in the cell and the known lack of polymerization ability of the CcmAI55A_mNG fusion, we expected the changes to occur mainly in the region of fast-moving molecules. Thus, to better account for this fraction of proteins in the molecules, we moved to an exposure time of 20 ms. Only a small shift in population sizes toward faster molecules or CcmA_mNG in the SQD analysis was shown by studying the difference between the two exposure times (Figures 4C,D and Table 2). Subsequently, we compared the CcmA_mNG fusion with the CcmAI55A_mNG fusion using the mean-squared displacement (MSD) analysis. In fact, MSD analysis of at least three independent SMT experiments with 20-ms exposure time demonstrated that the CcmAI55A_mNG derivative moved faster than CcmA_mNG (Figure 4C). To answer the question of which of the subpopulations might represent the polymeric and non-polymeric fractions, we compared the dynamics of CcmA_mNG and CcmAI55A_mNG using the provided apparent diffusion (AppD) analyses of the SMT software. AppD determines the diffusion constants based on the time-averaged MSD. Figure 4F shows the estimated population and diffusion coefficients (Table 3) illustrated by bubble plots. While 42.3% of the CcmA_mNG molecules showed static behavior, only 19% of the molecules in the CcmAI55A_mNG variant belonged to this fraction. Accordingly, the proportion of fast-moving molecules of CcmAI55A_mNG increased to 49.3%. With 34.5% for CcmA_mNG and 31.7% for CcmAI55A_mNG, almost no difference was observed in the slow-moving fraction. Based on these results, we can assume that the polymeric, and thus presumably functional, state of CcmA is represented by the static population.

**TABLE 2:** Summary of CcmA_mNG single-molecule dynamics acquired with 20- and 50-ms exposure time according to squared displacement analysis (SQD).

Strain	G27_*ccmA*_*mNG* (20 ms)	G27_*ccmA*_*mNG* (50 ms)
**SQD**		
Static fraction	33.5 ± 0.001	40.6 ± 0.002
Slow-mobile fraction (%)	50.1 ± 0.001	46.1 ± 0.002
Mobile fraction (%)	16.5 ± 0.000	13.4 ± 0.002
Static *D* (μm^2^ s^–1^)	0.015 ± 0	0.005 ± 0
Slow-mobile *D* (μm^2^ s^–1^)	0.07 ± 0.002	0.027 ± 0.002
Mobile *D* (μm^2^ s^–1^)	0.62 ± 0.007	0.32 ± 0.007

**TABLE 3:** Summary of CcmA_mNG single-molecule dynamics according to mean-squared displacement (MSD) and apparent diffusion (APPD) analysis.

Strain	G27_ccmA_mNG	G27_*ccmA_I55A_mNG*
**MSD**		
#Tracks	2785	2192
*D xy* (μm^2^ s^–1^)	0.026	0.114
**APPD**		
Static fraction	42.3 ± 0.000	19.0 ± 0.001
Slow-mobile fraction (%)	34.5 ± 0.001	31.7 ± 0.001
Mobile fraction (%)	23.1 ± 0.001	49.3 ± 0.001
Static *D* (μm^2^ s^–1^)	0.008 ± 0	0.008 ± 0
Slow-mobile *D* (μm^2^ s^–1^)	0.076 ± 0.002	0.076 ± 0.002
Mobile *D* (μm^2^ s^–1^)	0.467 ± 0.007	0.467 ± 0.007

### Csd7_mNG Forms Extensive Filamentous Structures Distributed Throughout the Cell With Distinct Membrane Association *in vivo*

**FIGURE 5:**
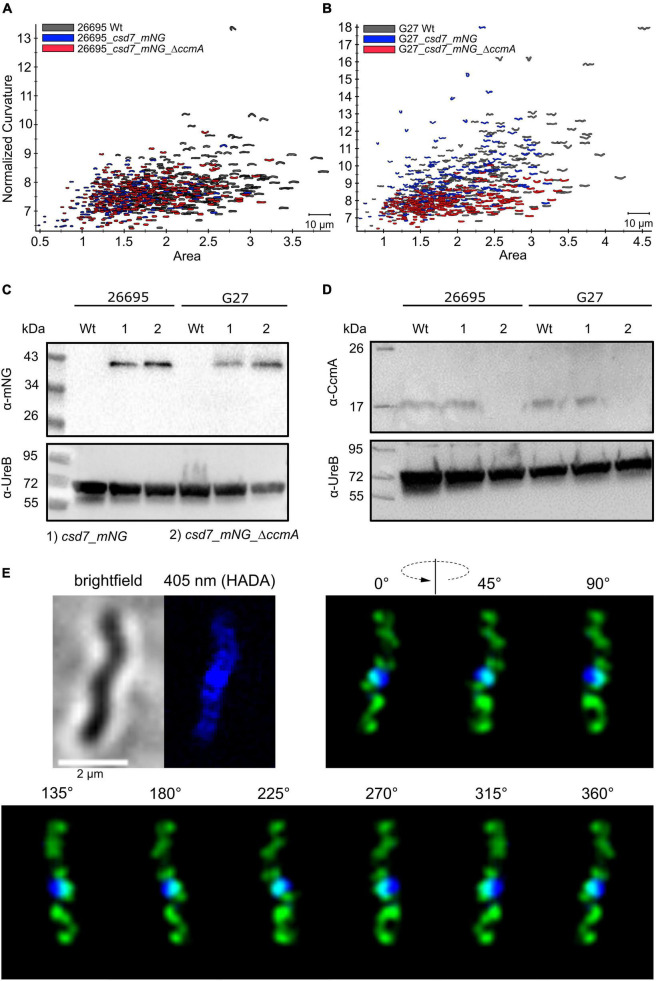
**(A,B)** Morphological comparison of the wild-type strains (26695, G27-gray) to the respective *csd7*_*mNG* (blue) strains and their bactofilin knockout variants (red) by principal component analysis. Cells are illustrated in a scatterplot according to their length (*x*-axis) and side curvature (*y*-axis). G27 wild-type, *n* = 253; G27_*csd7*_*mNG*, *n* = 251; G27_*csd7_mNG_ΔccmA*, *n* = 258; 26695 wild-type, *n* = 278; 26695_*csd7_mNG*, *n* = 272; 26695_*csd7_mNG_ΔccmA*, *n* = 270. Western blot depicting stable fluorescent fusions of mNG with Csd7 in 26695 and G27 **(C)** and verifies the correct bactofilin deletion in Csd7_mNG strains **(D)**. The usage of urease B-antibody functions as a loading control for each western blot. **(E)** Structured illumination microscopy of the Csd7_mNG fusion protein in *H. pylori* G27. Exemplary cell with Csd7_mNG (green) and newly incorporated peptidoglycan visualized by pulse labeling (20 min) with HADA (blue). Cells were acquired *via* Z-stack and projected to a 3D-image. Montage shows cells from different angles. Csd7_mNG fusion forms membrane-associated filamentous structures along with the cell. Scale bar = 2 μm.

**FIGURE 6:**
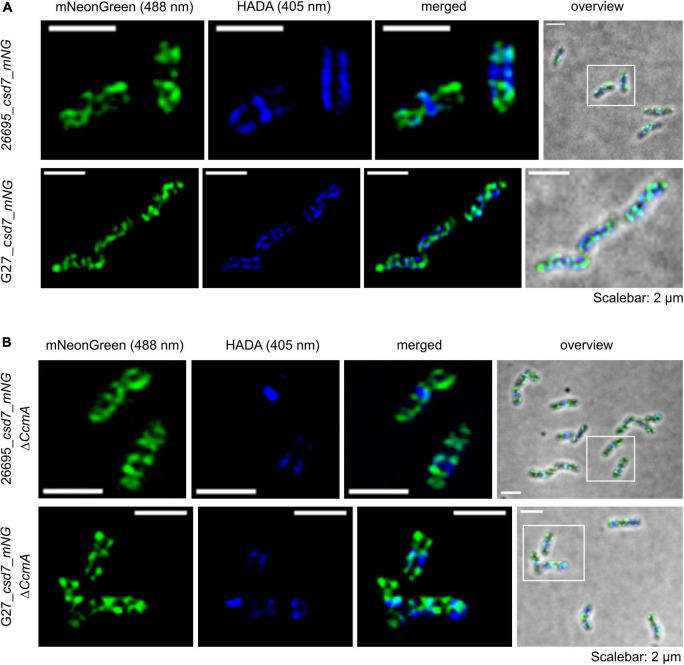
Structured illumination microscopy of the Csd7_mNG fusion protein in *H. pylori* G27 and 26695 under wild-type conditions **(A)** and bactofilin deletion background **(B)**. Exemplary 26695 and G27 cells with Csd7_mNG (green) and newly incorporated peptidoglycan visualized by pulse labeling (20 min) with HADA (blue). Cells were acquired *via* Z-stack and projected by the average intensity. Scale = 2 μm. The signal was located at the membrane with a predominant localization at the cell termini. The comparison revealed no altered signal localization pattern (microscope settings mNG: 488 nm laser, 10.0%, exp. time: 20 ms; HADA: 405 nm laser, 10.0%, exp. time: 50 ms; 3 rotations, EMCCD gain: 50.0).

Next, we also wanted to characterize the potential interacting partner Csd7 of CcmA and additionally investigate this suggested interaction of Csd7 and CcmA from the other perspective. The exact function of the membrane protein Csd7 is still unknown. Therefore, we constructed a Csd7_mNG fusion both in wt strains G27 and 26695 and the corresponding CcmA deletions. Considering that the loss of Csd7 also leads to altered cell morphology (Yang et al., 2019), we verified the functionality using the quantitative cell shape analysis according to the analysis of the CcmA_Gfp fusion strains performed. For this purpose, the CellTool software was used (Pincus and Theriot, 2007) and the results were visualized as cell contours on a coordinate plane (Figures 5A,B). Because the spiral morphology is more pronounced in strain G27, the differences among wild-type cells (gray), the Csd7_mNG fusions (blue), and the bactofilin-deficient derivatives (red) are more apparent in this strain than in the less curved strain 26695. However, in both cases, the Csd7_mNG fusion strains (blue) had similar curvatures to the wild type (gray) and were only slightly shorter, reflecting a rather minor influence of the protein fusion. Bactofilin-deficient cells (red) exhibited the expected straighter morphology, demonstrating the successful introduction of the bactofilin deletion in the Csd7_mNG fusion strains. In addition, western blot analysis demonstrated that the Csd7 protein fusion was neither degraded nor resulted in altered CcmA expression in either strain (Figures 5C,D). Detailed *in vivo* localization analyses using structured illumination microscopy (SIM) images of the Csd7_mNG fusion in both strains revealed extensive filamentous structures with clear membrane association (Figures 5E, 6A). Subjective observation of these structures gave the impression of a perpendicular arrangement. Relatively abundantly, in exponentially growing cells appearing to be close to division, a filamentous structure is seen spiraling along the membrane from the pole to the septum. To illustrate this result, an exemplary cell of strain G27 is shown as a projected 3D image and as a movie (Figure 5E and Supplementary Movie 2).

We next examined the potential colocalization of Csd7_mNG with the areas of increased peptidoglycan incorporation by pulsed incubation with the fluorescently labeled D-alanine derivative HADA (3-[[(7-hydroxy-2-oxo-2H-1-benzopyran-3-yl)carbonyl]amino]-D-alanine). Initial qualitative visual inspection of the Csd7_mNG fusion in both wt strains gave the impression that both signals were often present in close proximity to each other but did not clearly overlap (Figures 5E, 6A), complicating interpretation of colocalization status. As with CcmA_Gfp, we therefore quantified the signals and used the Pearson correlation coefficient to measure the statistical relationship. Pearson correlation analysis (PCC) yielded a Pearson correlation coefficient of *r* = 0.499 and *r* = 0.544 for strains G27_csd7_mNG and 26695_csd7_mNG, respectively. These results suggest that about 50% of the two signals overlap, so that at least some colocalization has occurred (Figure 2C). Interestingly, the localization of Csd7_mNG does not appear to be altered in the CcmA-deficient strains from either wt (Figure 6B). Moreover, the Pearson correlation coefficients of the CcmA-deficient strains were *r* = 0.609 (G27_cds7_mNG_ΔccmA) and *r* = 0.575 (26695_cds7_mNG_ΔccmA), giving approximately the same or slightly higher probability of colocalization with newly synthesized peptidoglycan (Figure 6B).

### The Absence of CcmA Does Not Change the Dynamics of Csd7_mNG

**FIGURE 7:**
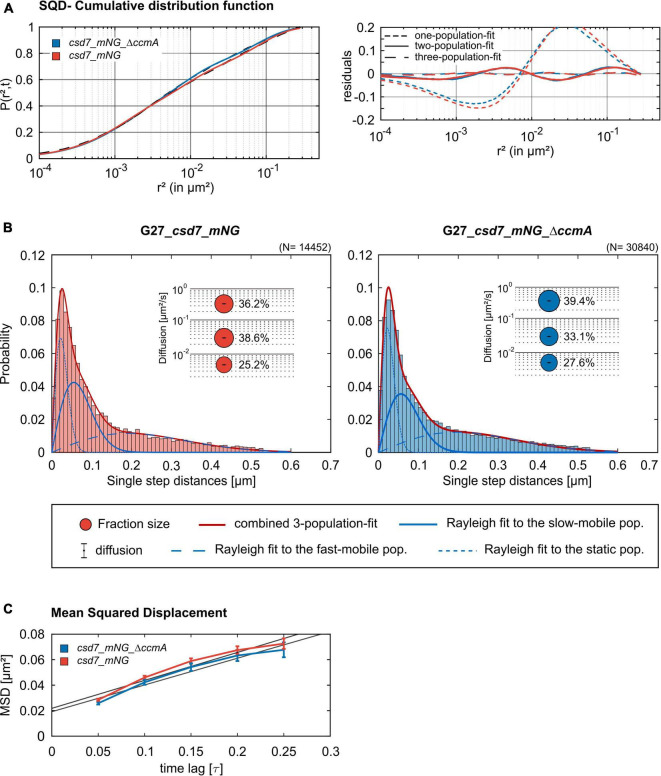
Single-molecule tracking analysis of the G27_*csd7*_*mNG* strain and the bactofilin-knockout strain. Data represent a biological triplicate with 95 cells, 2,056 tracks for G27_*csd7*_*mNG* and 110 cells, 4,326 Tracks for G27_*csd7*_*mNG_ΔccmA*. The movies were acquired with an exposure time of 50 ms per frame. **(A)** The cumulative distribution function (left panel) indicates the best fit for three populations. Residual analysis (right panel) is a quality indicator of the SQD analysis: the difference between the measured data (colored line) and modeled data (“zero” line) confirms three population assumptions. According to the data, we detected marginal effects of the bactofilin deletion in G27. With diffusion coefficients barely changing, a small fraction of the slow-mobile population shifts to the mobile and static populations. **(B)** Squared displacement analysis (SQD) of Csd7_mNG in G27 wt and CcmA deletion background. Diagrams display the probability for each detected jump distance. Bubble plots show the estimated population and diffusion coefficients as identified by non-simultaneous SQD curve fitting. N, number of steps. **(C)** Mean-squared displacement curves show a general diffusion regardless of subdiffusion. According to the MSD, the deletion of CcmA did not affect the overall diffusion of Csd7.

Next, we addressed the dynamics of Csd7 both in the presence and in the absence of CcmA using the single-molecule tracking (SMT) approach with an exposure time of 50 ms. As such, the SQD analysis of Csd7_mNG resulted in (at least) three populations of diffusive molecules (Figure 7A) as identified by residual analysis (Figure 7A). According to SQD analysis, 36.2% of Csd7_mNG molecules were assigned to the mobile fraction, with a diffusion coefficient of 0.36 μm^2^/s, 38.6% of molecules showed an intermediate average diffusion coefficient of 0.03 μm^2^/s, and 25.2% comprised the static fraction with *D* = 0.005 μm^2^/s (Figure 7B left panel and Table 4). Only minor changes were observed with respect to *ccmA* deletion (Figure 7 and Table 4). In strain G27, the main change was observed in the slow mobility population. However, these data show that the size of the slow-moving population of Csd7 decreases very slightly in the absence of the potential interaction partner CcmA. In each of the other two populations, there was a minimal increase. Even smaller variation was noted in the SQD analysis in the same constellation of strain 26695 (Table 5 and Supplementary Figure 6). Likewise, further mean-squared displacement (MSD) analysis revealed that the deletion of the bactofilin had no effect on the overall diffusion behavior of the Csd7_mNG in both strains (Figure 7C and Supplementary Figure 6B). Thus, including all single-molecule tracking data, it can be noted that the absence of bactofilin CcmA has only a marginal effect on the dynamics and diffusion behavior of membrane protein Csd7 in strains G27 and 26695.

**TABLE 4:** Summary of Csd7_mNG single-molecule dynamics in *H. pylori* G27.

Strain	G27*_csd7_mNG*	G27_*csd7_mNG_*Δ *ccmA*
**MSD**		
#Tracks	1397	2989
*D xy* (μm^2^ s^–1^)	0.052	0.053
**SQD**		
Static fraction	25.2 ± 0.001	27.7 ± 0.001
Slow-mobile fraction (%)	38.6 ± 0.001	33.1 ± 0.001
Mobile fraction (%)	36.2 ± 0.001	39.2 ± 0.001
Static *D* (μm^2^ s^–1^)	0.005 ± 0	0.005 ± 0
Slow-mobile *D* (μm^2^ s^–1^)	0.03 ± 0	0.03 ± 0
Mobile *D* (μm^2^ s^–1^)	0.36 ± 0.001	0.38 ± 0.001

**TABLE 5:** Summary of Csd7_mNG single-molecule dynamics in *H. pylori* 26695.

Strain	26695*_csd7_mNG*	26695_*csd7_mNG_*Δ *ccmA*
**MSD**		
#Tracks	1699	1754
*D xy* (μm^2^ s^–1^)	0.044	0.037
**SQD**		
Static fraction	25.5 ± 0.001	25.5 ± 0.001
Slow-mobile fraction (%)	41.6 ± 0.001	42.3 ± 0.001
Mobile fraction (%)	32.9 ± 0.001	32.2 ± 0.001
Static *D* (μm^2^ s^–1^)	0.005 ± 0	0.005 ± 0
Slow-mobile *D* (μm^2^ s^–1^)	0.03 ± 0	0.03 ± 0
Mobile *D* (μm^2^ s^–1^)	0.35 ± 0.001	0.37 ± 0.001

## Discussion

Recent studies shed light on a novel mechanism of bacterial shape generation in the helically twisted epsilon-proteobacterium *H. pylori.* This novel mechanism has been shown to rely on the activity of several PG-modifying endopeptidases and non-enzymatic factors such as the membrane-spanning proteins Csd5 and Csd7 (Blair et al., 2018; Yang et al., 2019), the membrane-associated bactofilin CcmA (Blair et al., 2018). Additionally, a set of coiled-coil-rich proteins (Ccrps) has been shown to influence the cell shape (Specht et al., 2011). The current working model assumes that at least some of these factors act in a dynamic complex to facilitate helical cell shape by local PG modifications (Taylor et al., 2020). Even though a relatively detailed image of this complex has been drawn in the literature, concrete information on dynamics and the interplay of these factors remain elusive. We focused on the *in vivo* localization and dynamics of the bactofilin CcmA of *H. pylori* and its putative interacting partners Csd7. In this context, we also addressed the question of whether both the number of CcmA clusters and CcmA dynamics is affected in the absence of a further putative interaction partner, Csd5. The ability of bactofilins to polymerize appears to be important for function, and polymerization is thought to be mediated *via* hydrophobic interactions between conserved hydrophobic residues in the core DUF583 domain (Vasa et al., 2015). Work on *T. thermophilus* bactofilin showed that the N-terminal region is involved in membrane binding (Deng et al., 2019). Our approach was based on strains expressing a *ccmA_gfp* fusion from the native *ccmA* locus under native expression conditions, resulting in a C-terminally tagged fusion protein. Although Gfp is almost two times the size of CcmA, we managed to obtain a fusion with an almost wild-type cell shape in both *H. pylori* strains 26695 and G27, as shown by 2D shape analysis. In previous studies, C-terminal bactofilin fusions had already been described as functional. For instance, the C-terminal eYfp fusion of BacA in *C. crescentus* still retained the ability to polymerize (Kühn et al., 2010), and in *A. biprosthecum*, the functional C-terminal BacA-mVenus fusion exhibited normal stalk formation (Caccamo et al., 2020). In addition to our Gfp fusions, we also generated a CcmA_mNG fusion carrying a linker sequence between the C-terminus and the fluorophore. Interestingly, this fusion appeared to even better restore the wild-type phenotype, indicating a potential role of the C-terminus in protein–protein interactions (S4). Both constructs localize similarly, as shown by the 3D SIM analysis, and also exhibit an analogous dynamic behavior. Moreover, the fact that in both strains, the CcmA fluorophore fusions localize to the membrane contributes to the fusion protein functionality as Taylor et al. (2020) demonstrated that CcmA mutants lacking polymerization do not associate with the membrane. This is also demonstrated by a comparison to our polymerization-deficient CcmAI55A_mNG fusion. For this point mutation, a limited function has already been described in a previous study (Taylor et al., 2020). As expected, the localization observed *in vivo* was detached from the membrane, thereby validating our obtained results. Analysis of the subcellular localization of CcmA with both fluorophores, which was performed using structural illumination microscopy (SIM) with exponentially growing cells, showed that CcmA was localized as small foci at the inner membrane in both strains, similar to published immunofluorescence data (Taylor et al., 2020). This confirmed the localization of CcmA previously observed by immunofluorescence (Taylor et al., 2020) *in vivo*. However, no clear localization preference for a specific curvature or defined pattern was observed, and CcmA was localized to both positive and negative curvatures. The same study found CcmA to be enriched in the areas of increased peptidoglycan (PG) synthesis, especially in the areas of negative Gaussian curvature and the major axis of the twisted helix (Taylor et al., 2020). Here, we used the fluorescently labeled D-Ala derivative HADA for probing PG synthesis (Kuru et al., 2019). We mainly observed signals at the septa and in sub-polar regions, which has also been described in the literature. Modified-D-Ala is incorporated into the pentapeptide through the action of PG transpeptidases where they are mainly found at the fifth position in *H. pylori* (Kuru et al., 2019). The HADA-labeling efficiency may be sensitive to local PG modifications and provides more knowledge about the septal growth zone. In the same context, only weak labeling of laterally dispersed PG insertion by fluorescently labeled D-Ala, as compared to other labeling approaches such as labeling the glycan backbone, has been reported for *H. pylori*. We found that CcmA frequently neighbored and partially overlapping with PG growth rings. Nevertheless, as indicated by Pearson correlation coefficient, this overlapping is neither positively nor negatively related. These growth rings can serve as landmarks to approximate the relative localization of CcmA in the further studies.

Interestingly, the recently described bactofilin homolog BacA from *A. biprosthecum* has been described as a recruitment platform and diffusion barrier (Giacomelli et al., 2022). In this organism, the bactofilin homolog BacA was demonstrated to interact with the PG-modifying enzyme SpmX to form pseudostalks. There, BacA presumably subsequently recruits other cell wall-modifying enzymes. In addition, BacA appears to retain SpmX and other factors at the base of the growing pseudostalk, preventing diffusion into the stalk. If we apply these findings to the shaping complex of *H. pylori*, a similar role of CcmA as a recruitment platform seems very likely. In this context, the interaction of CcmA with other cell shape-determining factors postulated in previous studies, such as Csd1/Csd2, Csd7, and Csd5, is also consistent. However, how these factors interact with each other and how this is spatially and temporally controlled is rather unclear, as the interactions were inferred from *in vitro* data such as immunoprecipitation and bacterial two-hybrid data. In addition, recent studies have changed the assumption that nucleotide-independent scaffold proteins of the cytoskeleton to which bactofilins belong constitute rigid scaffolds (Giacomelli et al., 2022). In *B. subtilis*, for example, the molecular function of DivIVA as a scaffolding platform undergoes modulation by other protein factors and is targeted to a specific molecular task that makes either a more stable complex dynamic or a more dynamic complex stable in response to protein–protein interactions (Feddersen et al., 2021). To answer the question of the dynamics of CcmA in living cells using a cell biology approach, we investigated this with single-molecule tracking using the SMT software (Rösch et al., 2018). In this context, we assume that if other shaping factors are controlled by CcmA, their diffusion behavior would change accordingly in the presence or absence of CcmA and *vice versa.*

By analyzing single-molecule steps using the squared displacement model (SQD) and the Gaussian mixture model, we identified at least three diffusive subpopulations for CcmA due to the limitation of the software. In both models, diffusion constants and population sizes were in the same range. We termed the slowest population static and the more mobile populations slow-mobile and fast-mobile fractions. Both models illustrate that the static and the fast-mobile fractions represent the largest and smallest populations, respectively.

Based on our previous knowledge of CcmA, we assume that the static fraction represents the sheet-like polymer structure and the fast-mobile fraction represents the freely diffusive units, whereas the intermediate fraction most likely represents an intermediate form of these two states. A comparison of CcmA with CcmAI55A under the same imaging conditions confirmed that the static fraction corresponds to the polymeric state, leading to the further conclusion that this fraction is the one required for cell shaping. Previous studies have proposed a speculative model in which the two non-enzymatic putative scaffold proteins, Csd5 and Csd7, and Csd1-3 and MurF, work together to promote helical twisting of the cell shape.

Previous studies postulated that the N-terminal and transmembrane domains of Csd5 and the integral membrane protein Csd7 interact with CcmA to be the part of a multiprotein transmembrane complex to connect the cytoplasm to the periplasm. However, according to our observations, CcmA molecules tend to diffuse only slightly more slowly in the absence of both putative interaction partners. Moreover, neither the absence of Csd5 nor Csd7 has any effect on the formation of CcmA clusters. Interestingly, the same tendency of Csd7 dynamics was observed in the absence of CcmA. However, a closer examination of the localization pattern of Csd7 using structured illumination microscopy (SIM) images of the generated Csd7_mNG fusion strain in both strain 26695 and G27 revealed a very different pattern from that observed in the CcmA fusion strains. As such, extensive filamentous structures were observed with clear membrane association and the impression of a perpendicular arrangement. Similar to CcmA, Csd7_mNG was found near PG growth rings. Interestingly, as indicated by Pearson correlation coefficient, the Csd7 fusion was lightly co-localized with the PG machinery, unlike CcmA. Since no direct interaction between Csd7 and CcmA has been found so far, we hypothesize that they might interact indirectly in the same dynamic complex. However, we have not observed major changes in the dynamics of CcmA in the absence of Csd7 or *vice versa*, so it seems more likely that the two act in different subcomplexes. As described previously, CcmA and Csd7 share the PG precursor synthase MurF as an interaction partner (Yang et al., 2019). MurF normally acts on ATP-driven synthesis of PG precursors in the cytoplasm and interacts directly with the elongasome *via* MurG (Favini-Stabile et al., 2013), MraY (White et al., 2010), and MreB (Mohammadi et al., 2007). Interestingly, four N-terminal transmembrane domains were identified for *H. pylori* MurF (Blair et al., 2018). Also, in agreement with our 3D-SIM data, it would be conceivable that Csd7 also prefers elongasome-like localization, whereas CcmA has more localized foci. While we did not find large changes in the dynamics of CcmA or Csd7, we did detect a slight tendency of both molecules to diffuse more slowly when putative interaction partners are absent. A possible explanation for this could also be that the proteins are uncoupled from the driving force of the main complex. Another possibility with respect to CcmA is that fewer molecules are released from the complex when interaction partners are absent. However, both ideas are highly speculative and require further investigation.

In summary, our work provides the first *in vivo* localization and dynamics of the bactofilin CcmA and its putative interaction partner Csd7 and thus makes an important contribution to the model of how CcmA might act within the postulated shapeosome complex. Based on our results, we can now also question whether CcmA could be a linker and thus possibly a regulatory factor between different complexes, as the interaction with both putative interaction partners seems to be temporally limited, if it occurs at all. Thus, this study provides further insight into the strategy of *H. pylor*i to regulate its helical cell shape, which is critical for its virulence.

## Materials and Methods

### Bacterial Strains and Growth Conditions

*Helicobacter pylori* strains were routinely cultivated on Dent blood agar in a microaerobic atmosphere as described earlier (Schätzle et al., 2015). Growth experiments were performed in Brucella broth with 5% fetal calf serum (BBF). All growth experiments were performed in triplicate and were repeated at least three times. *E. coli* strains were grown aerobically at 37°C in Luria-Bertani media. When needed, growth media were supplemented with 50 μg/l ampicillin, 20 μg/l kanamycin, or 20 μg/l chloramphenicol, respectively. Ellipsoid testing was performed according to the manufacturer’s instructions. For visualization of cell wall synthesis, we utilized the fluorescently labeled D-amino acid derivative HADA according to Kuru et al. (2019). HADA was added to a final concentration of 0.5 mM, and the samples were incubated under a micro-aerophilic atmosphere for 30 min at 37°C, 150 rpm. Subsequently, two parts of ice-cold ethanol were added and incubated for 10 min on ice. Cells were washed three times in PBS before mounting and imaging.

### Molecular Cloning and *H. pylori* Mutagenesis

Cloning was performed in *E. coli* according to standard protocols. Restriction and modifying enzymes (New England Biolabs, United States) were used according to the manufacturer’s instructions. Plasmids were isolated from *E. coli* with the QIAprep Spin MiniPrep Kit (QIAGEN, Germany), E.Z.N.A. Plasmid DNA Mini Kit (Omega Bio-Tek, United States), and Monarch Plasmid Miniprep Kit (NEB). The isogenic *H. pylori csd5* and *csd7* deletion mutants were constructed as described earlier (Waidner et al., 2009; Schätzle et al., 2015). Briefly, the resistance marker gene (kan) was fused to upstream and downstream DNA regions of mutagenized genes using a modified version of the megaprimer polymerase chain reaction (PCR) protocol (Sarkar and Sommer, 1990) and primers listed in Table 6. Subsequently, marker exchange mutagenesis of *H. pylori* was performed according to the standard procedures (Leying et al., 1992). *H. pylori* mutants carrying the resistance genes inserted into the chromosome were selected by growth on Dent blood agar containing kanamycin at concentrations of 20 mg/l. The correct insertions were verified by PCR and sequencing. Notably, downstream of csd5 is aroE and encodes shikimate dehydrogenase, which plays an essential role in basic metabolism. Since deletion of Csd5 did not result in growth restriction, a polar effect on the aroE gene is not likely. Csd7 is localized at the end of an operon. The isogenic *H. pylori ccmA_gfp* strain was generated using the double crossover integration of the pRDX-C (Smeets et al., 2000) in which the *rdxA* coding regions were replaced by the *ccmA_gfp* fusion and the coding region of the downstream gene. Construction of ccmA_mNG fusion was performed by replacing Gfp from already existing pRDX-C_ccmA_gfp_1541 plasmid with mNG *via* vector-PCR and Gibson assembly (New England Biolabs). CcmA mutant I55A was separately inserted in pRDX-C_ccmA_mNG according to the published method (Edelheit et al., 2009).

**TABLE 6:** List of strains, primers, and plasmids used in this study.

Strains: *H. pylori*			

Name	Description	Construction	References
26695	wt		Tomb et al., 1997
G27	wt		Baltrus et al., 2009
26695_*ccmA_KM*	26695 *ccmA::Pneo*		Holtrup et al., 2019
G27_*ccmA_KM*	G27 *ccmA::Pneo*		Holtrup et al., 2019
26695_*ccmA_gfp*	26695 wild type containing a *gfp* fused to *ccmA* and a cat resistance cassette at the original locus	Natural transformation with the construct pRDX-C_ccmA_gfp_1541	Specht, 2013
26695_*ccmA_gfp*Δ*csd5*	26695*_ccmA_gfp* with *csd5* replaced by a Kanamycin resistance cassette	Natural transformation of 26695_ccmA_gfp with the cross-over PCR-product aroE_KanR_HP1251	This study
26695_*ccmA_gfp*Δ*csd7*	26695*_ccmA_gfp* with *csd7* replaced by a Kanamycin resistance cassette	Natural transformation of 26695_ccmA_gfp with the cross-over PCR-product csd7-3&apos_KanR_csd7-5&apos	This study
G27_*ccmA_gfp*	G27 wild type containing a *gfp* fused to *ccmA* and a cat resistance cassette at the original locus	Natural transformation with the construct pRDX-C_ccmA_gfp_1541	Specht, 2013
G27_*ccmA_gfp*Δ*csd5*	G27_*ccmA_gfp* with *csd5* replaced by a Kanamycin resistance cassette	Natural transformation of G27_ccmA_gfp with the cross-over PCR-product aroE_KanR_HP1251	This study
G27_*ccmA_gfp*Δ*csd7*	G27_*ccmA_gfp* with *csd7* replaced by a Kanamycin resistance cassette	Natural transformation of G27_ccmA_gfp with the cross-over PCR-product csd7-3&apos_KanR_csd7-5&apos	This study
26695_*csd7*_*mNG*	26695 wild type containing a *mNG* c-terminally fused to *csd7* and a cat resistance cassette at the original locus		This study
26695_csd7_mNG_Δ*ccmA*		Natural transformation of 26695 Wt with the PCR-product *1543-1542::KanR-1541*	This study
G27_csd7_mNG	G27 wild type containing a *mNG* c-terminally fused to *csd7* and a cat resistance cassette at the original locus		This study
G27_csd7_mNG_Δ*ccmA*		Natural transformation of G27 Wt with the PCR-product *1481-1480::KanR-1479*	This study
G27_ccmA_mNG	G27 wild type containing a *mNG* c-terminally fused to *1480* and a chloramphenicol resistance cassette at the original locus	Natural transformation with the construct pRDX-C_*ccmA*_*mNG* (CM)	This study
G27_ccmAI55A_mNG	G27_*ccmA_mNG* with replaced isoleucine to alanine at position 55	Natural transformation with the construct pRDX-C_*ccmAI55A*_*mNG* (CM)	This study

**Strains: *E. coli***			

**Name**	**Description**		**References**

DH5α	*E. coli* K12 derivate, *fhuA2 lac(del)U169 phoA glnV44*Φ*80&apos lacZ(del)M15 gyrA96 recA1 relA1 endA1 thi-1 hsdR17.* Used for molecular cloning		Bethesda Research Laboratories
**Plasmids**			
**Name**	**Relevant characteristics**		**References**
pRDX-C	pBC-SK containing a chloramphenicol resistance cassette flanked 5′ and 3′ by *rdxA* sequences		Smeets et al., 2000
pRDX-C_ccmA_gfp	pRDX-C where the first *rdxA* cassette was changed for *ccmA_gfp*		This study
pRDX-C_csd7_mNG(26695)	Plasmids for *csd7* with C-terminal fused *mNG* for integration at the original locus		This study
pRDX-C_csd7_mNG(G27)			This study
pRDX-C_ccmA_mNG	Integration plasmid for *ccmA_mNG* for integration at the original locus		This study
pRDX-C_ccmAI55A_mNG	Integration plasmid for replacing *ccmA* with the point mutated *ccmAI55A*, fused to *mNG*		This study
KanR_aroE_for	TAT TTT ACT GGA TGT AAT TGT TTT AGA GAA TAA TGA AAT TAA AAT CTT TTG GGG	Construction of Δ*csd5* strains	This study
aroR_mid_rev	CCG CAT TCA AGC GCG ATA CC	Construction of Δ*csd5* strains	This study
HP1251_KanR_rev	GTG ATA TTC TCA TTT TAG CCA TTC CTA CCC TCA ACG C	Construction of Δ*csd5* strains	This study
HP1251_mid_for	ATT AGT GGT GGC GGG TTT CC	Construction of Δ*csd5* strains	This study

**Primer**			

**Name**	**Sequence**	**Usage**	**References**

csd7_5′_Kan_for	TAT TTT ACT GGA TGA ATT GTT TTA GAG CTC AAA TAG GAA TAG CTA AAG	Construction of Δ*csd7* strains	This study
csd7_5′_rev	ACA CCC TGT GCC TGT GGT AG	Construction of Δ*csd7* strains	This study
csd7_3′_for	CAG TGC ATG CCA ATT CC	Construction of Δ*csd7* strains	This study
csd7_3′_Kan_rev	GTG ATA TTC TCA TTT TAG CCA TCT ACA ACC TAA TCA TTG CCT	Construction of Δ*csd7* strains	This study
KanR_for	ATG GCT AAA ATG AGA ATA TCA C	Amplification of the *kan* resistance cassette	This study
KanR_rev	CTA AAA CAA TTC ATC CAG TAA AAT A	Amplification of the *kan* resistance cassette	This study
pRDXc_csd7_xbaI_for	GAG TCT TAT AAA GTT CTA GAA TGA ATT TTT ATC AAA AAA T	Amplification of csd7	This study
csd7_EcoRI_mNG_rev	TCC TCG CCC TTG CTC ACC ATT CCG AAT TCA ATT TGA TGT TCC AAA CGC C		This study
mNG_for	ATG GTG AGC AAG GGC GA	Amplification of *mNeonGreen*	This study
mNG_rev	TTA CTT GTA CAG CTC GTC CA		This study
mNG_692-711_for	TGG ACG AGC TGT ACA AGT AA	Amplification of *cat-flhb* from pRDX-C_flhb	This study
pRDX-C_flhb_kpnI_rev	GGG AAC AAA AGC TGG GTA CCT GAC TAA ACA AGA AGT TAA G		This study
csd7_pRDX-C_xbaI_rev	ATT TTT TGA TAA AAA TTC ATT CTA GAA CTT TAT AAG ACT C	Vector PCR of pRDX-C	This study
flhb_pRDX-C_kpnI_for	CTT AAC TTC TTG TTT AGT CAG GTA CCC AGC TTT TGT TCC C		This study
mNeoVenChe_pRDX-C_for	TGG ACG AGC TGT ACA AGT AAG GAT CCC CCG GGC TGC AGG A	Vector-PCR of pRDX-C_CcmA_gfp_1541 to replace gfp to mNG	This study
1542-mNeoVenChe-ApaI_rev	TCC TCG CCC TTG CTC ACC ATT CCG GGC CCT TTA TTT TCA ATT TTC TTT TCT TGC TCA TTG ATT		This study
CcmA_I55A_for	ATT CTA AAA GCA CGG TGG TGG CCG GAC AAA CCG GCT CGG TAG	Site-directed mutagenesis in CcmA at position 55 (I to A)	Taylor et al., 2020
CcmA_I55A_rev	CTA CCG AGC CGG TTT GTC CGG CCA CCA CCG TGC TTT TAG AAT		Taylor et al., 2020

Csd7_mNG fusion constructions were conducted by amplifying the *csd7* gene from the respective chromosomal wild-type DNA and *cat-flhb* from pRDX-C_flhb (*rdxAII* was replaced with *flhb*) and were integrated with mNG into pRDX-C by Gibson assembly (New England Biolabs) according to the manufacturer’s manual. *H. pylori* strains 26695 and G27 were naturally transformed with the resulting plasmids, creating a C-terminal fusion of the *csd7* gene with mNG at the original locus.

### Immunoblotting

About 1 ml of exponentially growing cells was pelleted and subsequently stored at –80°C until usage. Equal amounts of total cell lysates were subjected to sodium dodecyl sulfate–polyacrylamide gel electrophoresis (SDS-PAGE) and transferred onto nitrocellulose membranes by western blotting (Hybond C Extra, GE Healthcare Life Sciences). For immunodetection, membranes were incubated with appropriately diluted primary antibodies and then probed with secondary antibodies conjugated with horseradish peroxidase. Bound antibodies were detected with enhanced chemiluminescent (ECL) detection reagents as substrates followed by incubation for 2 min and chemiluminescence detection with ChemiDoc MP System (Bio-Rad).

### Microscopy and Image Analysis

Phase-contrast images were obtained using an Olympus AX-20 microscope (objective: 100x/NA 1.30, oil, Ph) and a CoolSNAP HQ2 (Photometrics) camera. For quantitative shape analysis, binary images were generated using Fiji (Schindelin et al., 2012) and subsequently analyzed using the CellTool software (Pincus and Theriot, 2007).

High-resolution images of *H. pylori* were acquired by a ZEISS ELYRA PS.1 system using multi-color SIM (SR-SIM Mode) with three different excitation wavelengths: (I) 488 nm line, 10% intensity, gain 50, 100 ms (Gfp), 20 ms, and 50 ms (mNG); (II):405 nm line, 10% intensity, gain 50, 50 ms (HADA), and an EMCCD camera (ANDOR Solis EMCCD). SIM reconstructions were processed using ZEN-Black by ZEISS. Processing was done in Fiji (Schindelin et al., 2012) version 1.52p. Quantitative analysis was done using workflows based on R-statistics and R-studio version 3.6.0 using the packages tidyverse and ggplot2 (Wickham et al., 2019). Signal correlation in SIM images was quantified by using Pearson and Manders colocalization coefficients in the Fiji Plugin JACoP (Bolte and Cordelières, 2006).

Clusters of tracks as identified by TrackMate (Tinevez et al., 2017) were identified using the R packages DBSCAN (Hahsler et al., 2019) and fpc (Hennig, 2020).

For obtaining single-molecule dynamic information, we used a slim-field set up on a customized Nikon Ti Eclipse microscope (objective: 100x/NA 1.49, oil). Samples were illuminated using a 488 nm diode at max 160 W/cm^2^ on the image plane to initially bleach most fluorophores and subsequently observe single-molecule tracks using a EMCCD camera (ImagEM X2, Hamamatsu, Japan) with an exposure time of 50 (Gfp) and 20 ms (mNG). The movies were processed using ImageJ2/FIJI to remove the initial bleaching phase. Single-molecule tracks obtained using u-track (Jaqaman et al., 2008) and cell meshes generated by using Oufti (Paintdakhi et al., 2016) were formatted and loaded in our custom software SMTracker (Rösch et al., 2018) for analysis of single-molecule behavior using SMMTrack (Schenk et al., 2017). Localization errors are determined by the software according to Michalet (2010).

## Data Availability Statement

The data supporting the findings of this study are included in the publication/Supplementary Material, further inquiries may be directed to the corresponding author.

## Author Contributions

SH performed the experiments, analyzed the data shown in Figures 1–3 and Supplementary Figures 1–4, and helped in writing of the manuscript. BM and SH developed the principal component analysis tool to visualize different cell shape populations. BM helped to set up the SIM. MG performed the experiments, analyzed the data shown in Figures 4–7, Supplementary Figures 5, 6, and Supplementary Movies 1, 2, and helped in writing of the manuscript. MS constructed the *Helicobacter pylori* CcmA_Gfp strains. BW devised the study, supervised all experiments, helped to analyze the data, and wrote the manuscript. All authors contributed to the article and approved the submitted version.

## Conflict of Interest

The authors declare that the research was conducted in the absence of any commercial or financial relationships that could be construed as a potential conflict of interest.

## Publisher’s Note

All claims expressed in this article are solely those of the authors and do not necessarily represent those of their affiliated organizations, or those of the publisher, the editors and the reviewers. Any product that may be evaluated in this article, or claim that may be made by its manufacturer, is not guaranteed or endorsed by the publisher.
