# Supplementary material for: Insights Into the Helical Shape Complex of Helicobacter pylori
Source: Front Microbiol. 2022 Aug 24;13:929194. doi: 10.3389/fmicb.2022.929194 (PMC9448923; doi:10.3389/fmicb.2022.929194)
Supplement: Supplementary file 1 [file Data_Sheet_2.PDF]

Supplementary Material

1 SUPPLEMENTARY DATA

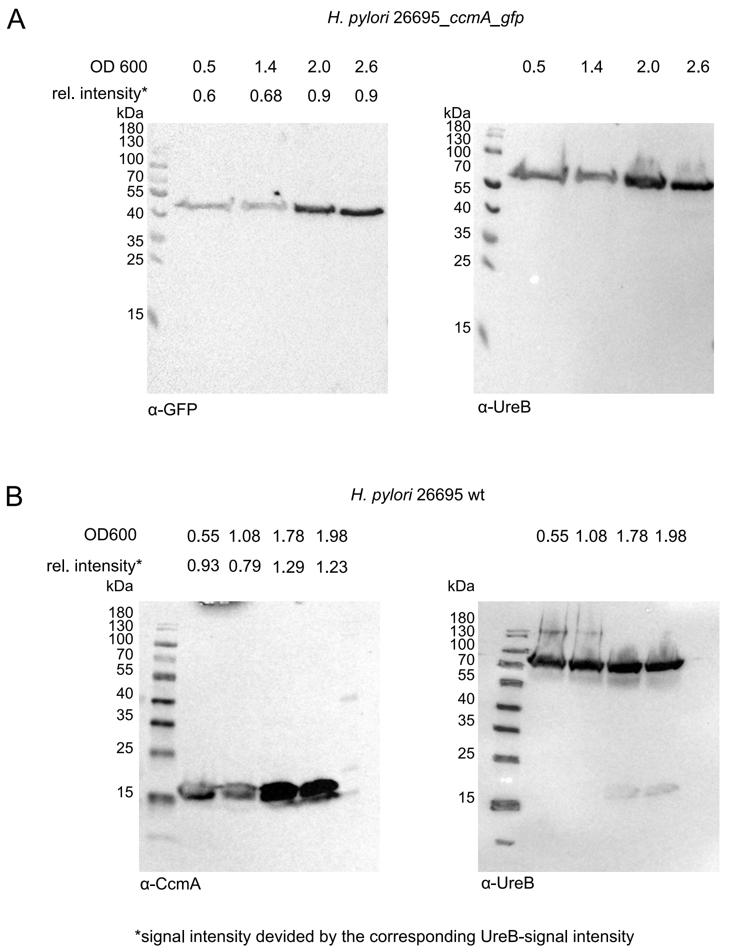

Figure S1. Semi-quantitative measurement of (A) CcmA_Gfp-levels in the *H. pylori* strain 26695_*ccmA_gfp* and (B) CcmA-levels in the 26695 wt strain at different stages of growth. *H. pylori* cells were initially normalized by their OD600 (upper lane) and analysed by SDS-PAGE and western-blotting, followed by immunodetection using gfp-specific monoclonar first-antibodies. As loading control, the nitrocellulose membrane was subsequently washed shortly in 0.5 M NaOH and redetected using UreB-specific monoclonal antibodies. Ratios of both signals are given in the second lane (rel. intensities).

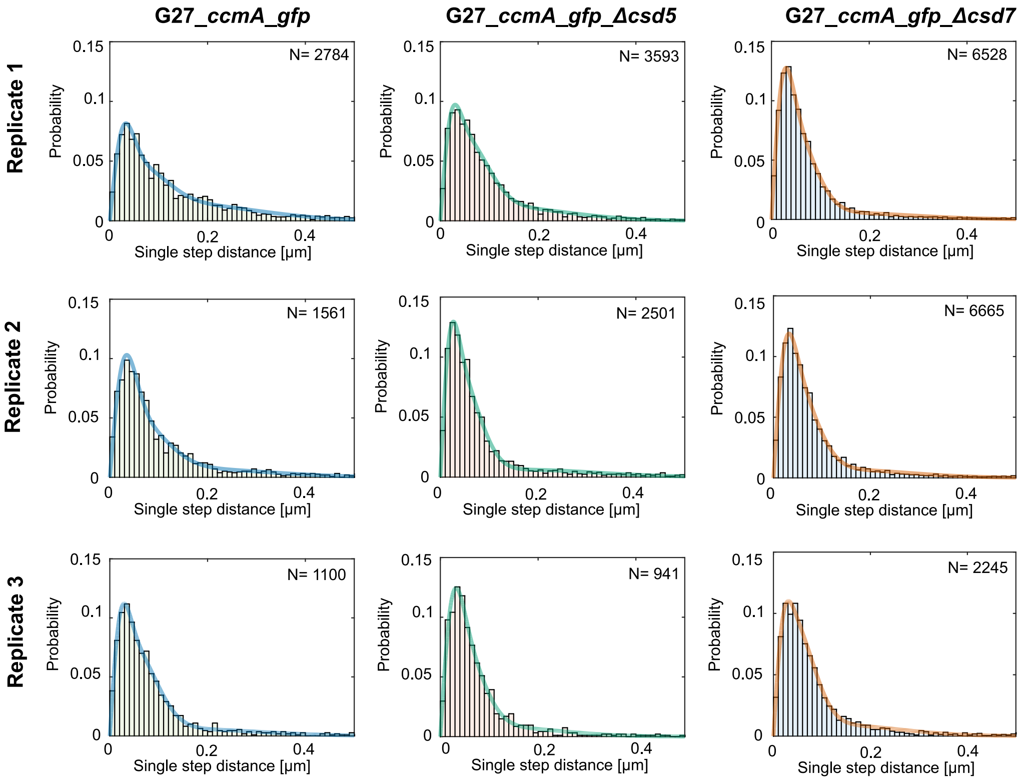

Figure S2. Probability densities of separate replicates of single-step distances of CcmA_Gfp in *H.pylori* 26695 wt, Δ*csd5* and Δ*csd7* backgrounds, as derived from slim-field bleaching microscopy movies by u-track and generated in the SMTracker software.

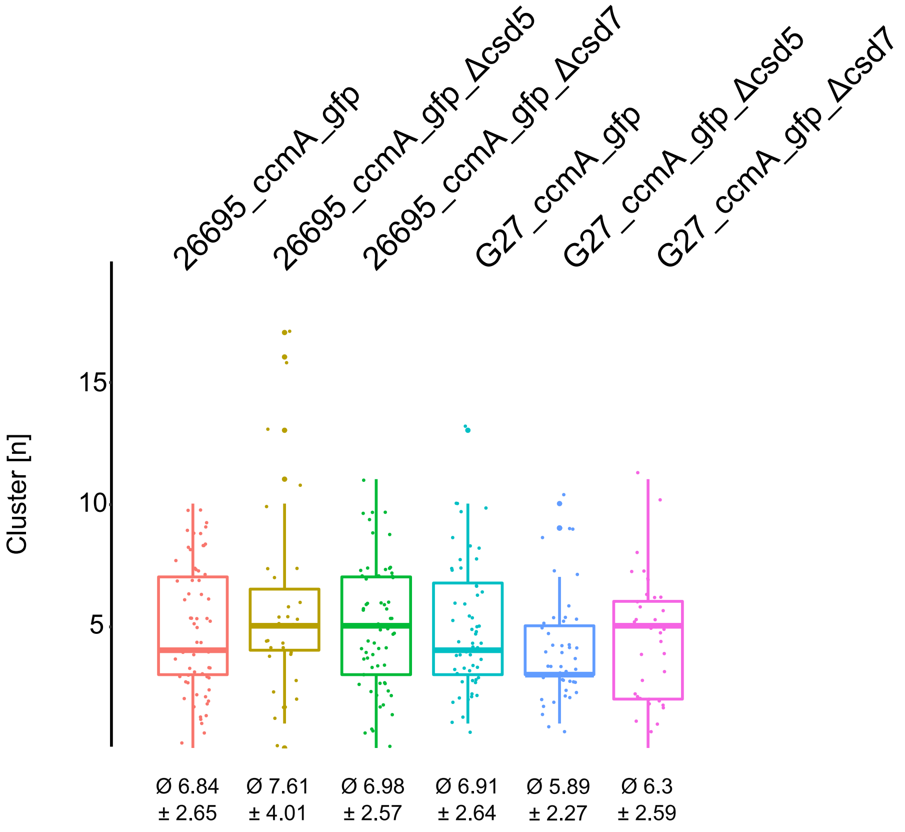

Figure S3. Number of clusters as identified by running the DBSCAN algorithm over track-coordinates identified by trackmate from slim-field bleaching microscopy movies. Clusters were analysed using the R packages fpc and dbscan. Parameters were set equal for all measurements.

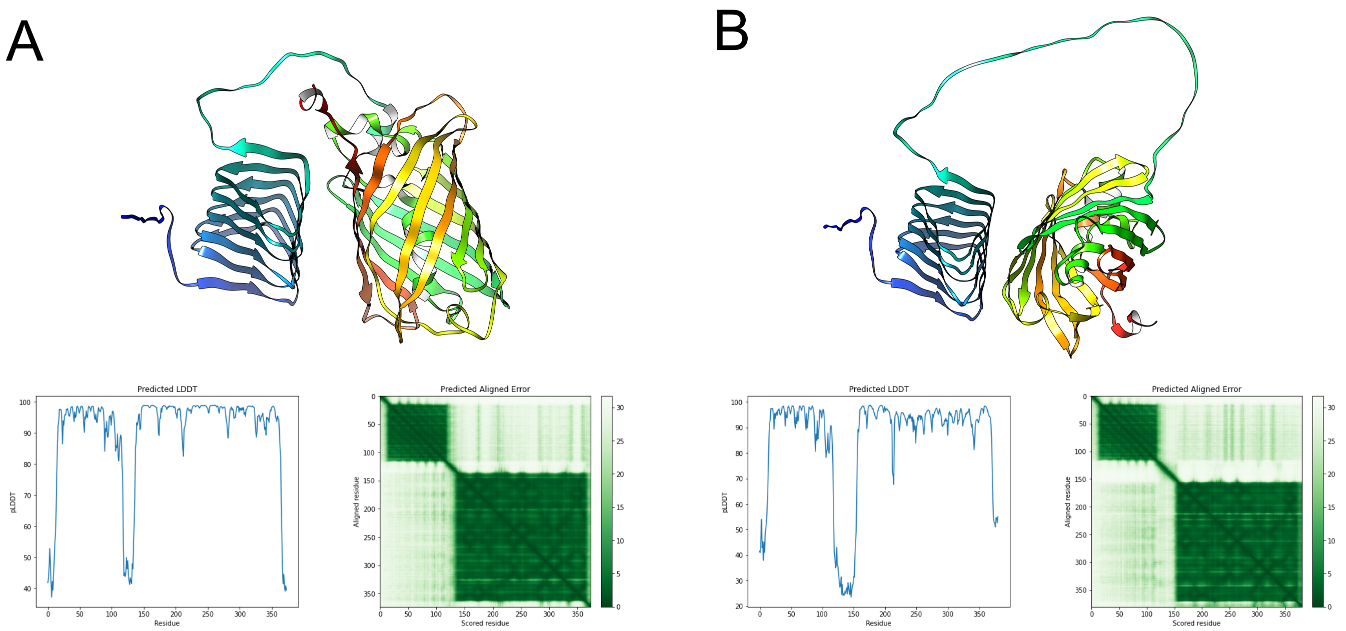

Figure S4. Structure predictions of CcmA_Gfp(A) and CcmA_mNG(B) as generated using the Alphafold-algorithm and visualized using UCSF-Chimera. Peptide chains are colored from blue through the rainbow spectrum to red in N- to C-terminal direction. Predicted local alignment errors and local Distance Difference Test results (LDDT) are given beyond the respective structures.

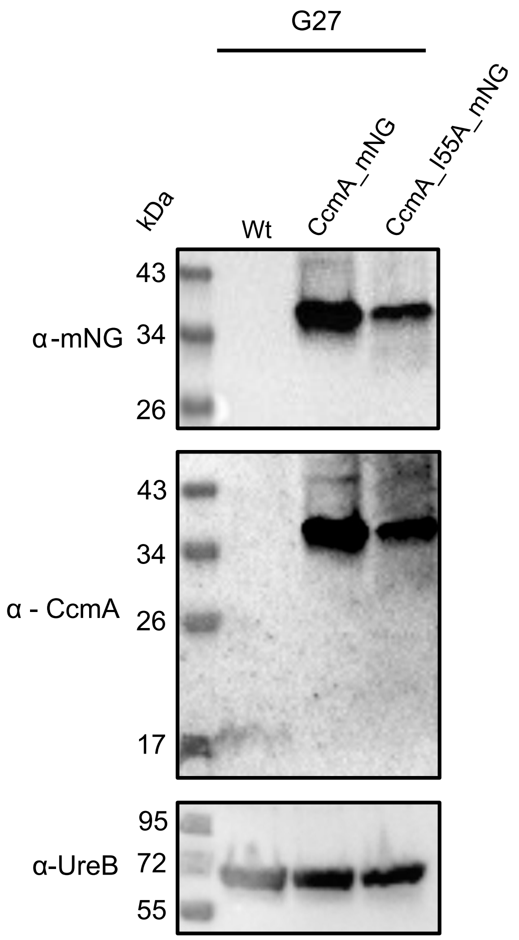

Figure S5. Western-blotting reveals that both, CcmA_mNG and CcmA_I55A_mNG are stable within the *H. pylori* strain G27. Samples of the strains G27 (wt), G27_ccmA_mNG and G27_ccmA_I55A_mNG were separated on a SDS-PAGE, blotted onto a nitrocellulose membrane and detected by using monoclonal anti-mNG antibodies. Subsequently, the membrane was stripped and redetected using CcmA and UreB antibodies respectively. Neither for CcmA_mNG, nor its mutated counterpart we observed additional signals beyond the signal of the expected protein size. Equal amounts of cell extract were applied for western-blotting, as indicated by the loading control UreB.

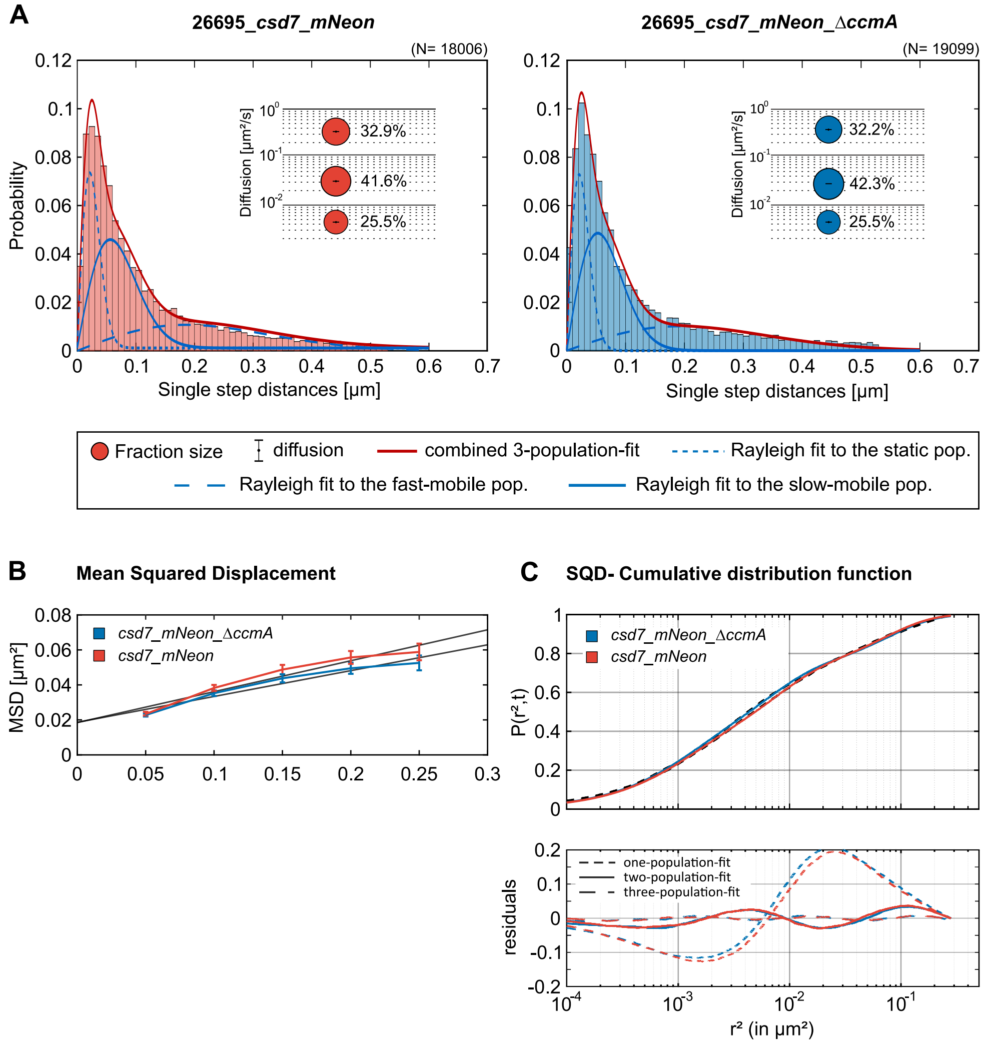

Figure S6. 2D-diffusion dynamics of Csd7_mNG in the *H. pylori* strain 26695 and 26695_Δ*ccmA*, as derived from slim-field bleaching microscopy movies. **A** Probability density distributions of single step distances and bubble-plots of identified subpopulation sizes. **B** Mean-squared displacement per time-lag, given in s. **C** Cumulative distribution function of squared-displacements, giving the probability of a molecule being in a certain radius at a certain time, as well as residuals of different subpopulation models to the distribution. the three subpopulations fit shows the smallest differences in the experimental data.
